# Rice nucleosome patterns undergo remodeling coincident with stress-induced gene expression

**DOI:** 10.1186/s12864-017-4397-8

**Published:** 2018-01-26

**Authors:** Qi Zhang, Dong-Ha Oh, Sandra Feuer DiTusa, Mangu V. RamanaRao, Niranjan Baisakh, Maheshi Dassanayake, Aaron P. Smith

**Affiliations:** 1 Department of Biological Sciences, Louisiana State University, Baton Rouge, LA 70803 USA; 2 School of Plant, Environmental, and Soil Sciences, Louisiana State University Agricultural Center, Baton Rouge, LA 70803 USA

**Keywords:** Nucleosome patterns, Phosphate starvation, Chromatin remodeling, Differential gene expression, MNase-seq, RNA-seq, Rice

## Abstract

**Background:**

Formation of nucleosomes along eukaryotic DNA has an impact on transcription. Major transcriptional changes occur in response to low external phosphate (Pi) in plants, but the involvement of chromatin-level mechanisms in Pi starvation responses have not been investigated.

**Results:**

We mapped nucleosomes along with transcriptional changes after 24-h of Pi starvation in rice (*Oryza sativa*) by deep sequencing of micrococcal nuclease digested chromatin and ribosome-depleted RNA. We demonstrated that nucleosome patterns at rice genes were affected by both *cis*- and *trans*-determinants, including GC content and transcription. Also, categorizing rice genes by nucleosome patterns across the transcription start site (TSS) revealed nucleosome patterns that correlated with distinct functional categories of genes. We further demonstrated that Pi starvation resulted in numerous dynamic nucleosomes, which were enhanced at genes differentially expressed in response to Pi starvation.

**Conclusions:**

We demonstrate that rice nucleosome patterns are suggestive of gene functions, and reveal a link between chromatin remodeling and transcriptional changes in response to deficiency of a major macronutrient. Our findings help to enhance the understanding towards eukaryotic gene regulation at the chromatin level.

**Electronic supplementary material:**

The online version of this article (10.1186/s12864-017-4397-8) contains supplementary material, which is available to authorized users.

## Background

Eukaryotic DNA must be condensed to fit into a small space within the nucleus. Studies on primary chromatin structure reveal that 146 bp of DNA wrap around a histone octamer consisting of each of two copies of H2A, H2B, H3 and H4 to comprise a nucleosome [1]. The formation of nucleosomes along DNA facilitates DNA compaction, however it makes the DNA inaccessible for important cellular processes including DNA replication, recombination, repair and transcription [2–5]. Hence, nucleosome distribution is not static but rather is modified in accordance with these processes. Studies in a variety of eukaryotes have identified two types of determinants of nucleosome occupancy: DNA sequence features, considered *cis*-determinants, such as GC content, and *trans*-determinants, including transcription factors and chromatin remodelers that modulate nucleosome placement, histone variant deposition, and histone post-translational modifications [6–13]. Previous studies have also demonstrated the presence of well-positioned nucleosomes adjacent to transcriptional start sites (TSS) of eukaryotic genes [6–8, 12–15]. In contrast, much less is understood regarding the dynamics of nucleosome patterns (i.e. positioning and occupancy) and their connections to transcriptional regulation.

In order to survive, organisms must respond rapidly and vigorously to environmental stress, such as low availability of nutrients. Phosphorus (P) is an essential nutrient as it is a structural component of nucleic acids and phospholipids, and is involved in the regulation of biological processes. Consequently, prolonged P starvation can lead to arrest of both growth and cell division [16]. To ensure coordination between growth and external P availability, organisms have evolved sophisticated sensing and signaling pathways. In budding yeast (*Saccharomyces cerevisiae*), a combination of transcriptional regulators and downstream targets comprising the PHO regulon modulate adaptive responses to deficiency of inorganic phosphate (Pi), a primary source of P [16]. Early studies examining the role of chromatin structure in transcriptional regulation showed that nucleosome remodeling also plays a role in modulating PHO regulon genes. Specifically, nucleosomes are evicted to expose the promoter region of the yeast *PHO5* secreted acid phosphatase gene in response to low-Pi conditions [17, 18]. More recent genome-wide studies on the remodeling of primary chromatin structure in response to environmental perturbation in yeast have shown a connection between global nucleosome dynamics and transcription activities [19, 20].

As sessile organisms, plants constantly encounter environmental challenges and must shape themselves for adaptation. Pi is one of the most limiting nutrients for plants due to its low solubility in soil and poor uptake efficiency [21]. Pi fertilizers are applied to maintain crop growth, but are mined from non-renewable sources, and over-fertilization of Pi can cause environmental problems, including eutrophication of waterways and hypoxia [22–25]. Understanding how plants respond to Pi limitation, and increasing Pi-use efficiency will aid in enhancing agriculture sustainability. Many studies have been carried out to investigate Pi starvation responses (PSRs) in plants. These studies have identified morphological and physiological responses aimed at enhancing Pi acquisition and recycling, as well as key regulators of these responses [26–29]. Transcript profiling studies have shown that transcriptional regulation plays an important role in modulating PSRs [30–33], and emerging data from our laboratory and others are indicating that chromatin-level mechanisms are also involved in regulating PSRs [34–36].

Studies of nucleosome occupancy and positioning in plants are limited compared to those in model animal species. Recent genome-wide studies in Arabidopsis [7, 37] and rice [13, 15] have shown nucleosome patterns in genic regions that are generally similar to other eukaryotes. Also similar to other species is that transcription is an important *trans*-determinant of nucleosome occupancy in plants [7, 8, 15, 38]. However, many questions remain regarding the particular determinants of nucleosome occupancy in plants and how they compare to other eukaryotes, as well as how environmental perturbation impacts nucleosome dynamics and the extent to which nucleosome remodeling is linked to changes in gene expression in plants or other systems. Herein, we report global nucleosome positioning and occupancy in rice (*Oryza sativa*), a staple crop that feeds billions world-wide, via deep sequencing of micrococcal nuclease-digested chromatin (MNase-seq), and demonstrate the impact of Pi starvation on nucleosome patterns. We also examined transcription activities by deep sequencing of ribosomal RNA-depleted RNA followed by quantification of intronic transcript abundance. By integrating information obtained from these assays, we reveal relationships among gene structure, gene function, nucleosome patterns, and transcription activities, including a significant correlation between nucleosome dynamics and changes in gene expression in response to limitation of a major essential nutrient.

## Results

### Nucleosome positioning and occupancy of rice genes

The goals of this study were to generate a high-resolution map of nucleosome patterns in rice, define the impact of environmental perturbation (via Pi starvation) on these patterns, and establish whether nucleosome dynamics in response to Pi starvation correlate with differential gene expression. Because a recent transcript profiling study in rice demonstrated that major changes in transcript abundance were detected beginning after 24 h of Pi starvation [30], and chromatin remodeling can happen within hours of environmental stress [19], we were interested in observing the changes in the organization of primary chromatin structure and exploring the correlations at this relatively early time point.

We generated a total of four MNase-seq libraries including two biological replicates from shoots (green tissue from the seedling) of plants grown in full nutrient hydroponic solution for 5 weeks with an additional 24-h of growth in either full nutrient hydroponic solution (control replicate 1 and 2, C1 and C2) or nutrient solution lacking phosphate (−Pi replicate 1 and 2, P1 and P2). Sequencing reads were mapped to the Michigan State University rice genome annotation release 7 (MSU7) [39]. We obtained on average 63 million uniquely mapped single-end reads with an average of 15× coverage of the rice genome for each library. To examine the reproducibility of the mapped reads, we calculated the Spearman’s correlation coefficient (SCC) of sequencing read abundance at each genomic position between C1 and C2 (SCC = 0.95) and P1 and P2 (SCC = 0.96) (Additional file 1: Fig. S1A). Considering the high reproducibility of mapped MNase-seq reads for each treatment, we generated profiles based on the average of two replicates for the ease of data presentation but kept replicates separated for data analyses.

**Fig. 1:**
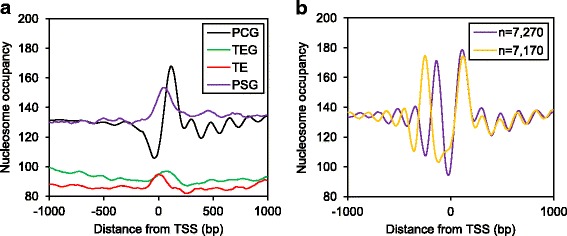
Nucleosome patterns across the transcription start site of rice genes. Regions 1000 bp upstream and downstream of the transcription start site (± 1000 bp TSS, or 5′ boundary of the element), were used to plot MNase-seq density under control conditions. (**a**) MNase-seq density for all rice genes across the transcription start site (TSS) under control conditions. PCG, protein-coding genes; TEG, transposable element related genes; TE, transposable elements; PSG, pseudo-genes, annotated genes that are neither expressed nor transposable elements. (**b**) MNase-seq density across the TSS of genes found to have −1 nucleosome at either −140 bp (7270 genes) or −250 bp (7170 genes) relative to the TSS

To determine nucleosome patterns of rice genes, we first separated all annotated genes into four categories according to the MSU7 genome annotation [39]: protein-coding genes (PCG), transposable elements (TE), transposable element-related genes (TEG), and ‘pseudogenes’ (PSG, i.e. annotated genes that are neither expressed nor transposable elements). Using representative gene models, the genes in each category were combined to generate nucleosome profiles for each group. For PCG, we observed several distinct nucleosome pattern features: First, evenly-spaced nucleosome arrays were observed downstream of the transcription start site (TSS; Fig. 1a); Second, a nucleosome-depleted region (NDR) was found immediately upstream of the TSS (Fig. 1a, Additional file 2: Fig. S2B); Third, higher nucleosome occupancy was found immediately downstream of the TSS and upstream of the transcription termination site (TTS) as compared to the remainder of the gene body (GB; Fig. 1a, Additional file 2: Fig. S2A and B). In contrast to PCG, neither evenly-spaced nucleosome arrays downstream of the TSS nor NDRs upstream of the TSS were found in the remaining three gene categories, indicating these features are signatures of rice genes poised for transcription (Fig. 1A, Additional file 2: Fig. S2A and B).

The nucleosome pattern for rice PCG that we observed is similar to findings from other studies, but highlights an apparent distinction among species—for example, evenly-spaced nucleosome arrays were found upstream of the TSS in genome-wide studies of human [12] and yeast [6], but not in rice [13, 15, and this study], Arabidopsis [7, 8], or *Tetrahymena thermophila* [40]. This may result from differences in either MNase-digestion strength [41], lengths of NDR regions and associated DNase I hypersensitivity sites [13], or sample complexity due to combined cell types. We hypothesized that diverse nucleosome patterns from subsets of genes were masking defined nucleosome arrays upstream of the TSS when combined in the single PCG nucleosome profile (Fig. 1a). Therefore, we analyzed nucleosome occupancy and positioning of all PCG separately to search for evenly-spaced nucleosome arrays. Interestingly, we identified two major subsets of PCG that each had nucleosome arrays not only downstream of the TSS but also upstream (Fig. 1b). These gene subsets had a well-defined −1 nucleosome (the first nucleosome upstream of the TSS) at either −140 bp (*n* = 7270) or −250 bp (*n* = 7170) relative to the TSS, with phased nucleosome arrays further upstream. The nucleosome arrays of these two groups of genes are out of phase with each other and thus mimic “destructive wave interference” when combined into a profile of all genes, masking the nucleosome peaks in the region upstream of the TSS (Fig. 1). Gene ontology (GO) term enrichment analysis [42] of the two subsets of genes did not yield any significantly enriched GO terms (false discovery rate (FDR) <0.05). This indicated no obvious functional link among the genes in each group, but rather the contribution of other determinants to the distinct nucleosome patterns.

### Rice nucleosome occupancy is affected by both *cis*- and *trans*-determinants

**Fig. 2:**
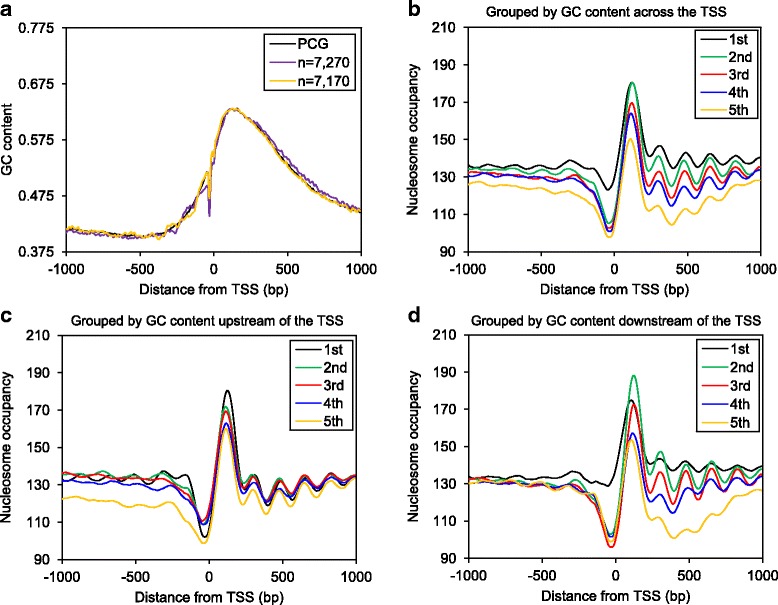
Correlations between GC content and nucleosome patterns. (**a**) GC content of genes across the TSS. (**b**) MNase-seq density of PCG grouped by their GC content across the TSS (± 1000 bp TSS), 1st lowest, 5th highest. (**c**) MNase-seq density of PCG grouped by their GC content at a window of 1000 bp upstream of the TSS, 1st lowest, 5th highest. (**d**) MNase-seq density of PCG grouped by their GC content at a window of 1000 bp downstream of the TSS, 1st lowest, 5th highest

To search for factors that contribute to nucleosome profiles of rice genes, we investigated GC content and transcription, which are known *cis*- and *trans*-determinants of nucleosome occupancy. It is widely reported that GC- and AT-rich sequences favor and disfavor nucleosome formation, respectively [9, 11, 12, 14, 15, 40]. However, a negative correlation between GC content and nucleosome occupancy was shown in Arabidopsis and rice when examining random fragments of genomic DNA [8], and few studies have examined the correlation between GC content and nucleosome occupancy across the TSS. To investigate GC content as a potential determinant of nucleosome occupancy in rice, we first plotted the GC content distribution of all PCG across the TSS, which broadly showed a pattern similar to nucleosome occupancy with a narrow trough immediately upstream of the TSS and a wide peak downstream of the TSS (Fig. 2a). Secondly, we plotted the GC content distribution of the −140 and −250 gene subsets separately and observed opposing oscillations of GC content upstream of the TSS, such that the GC content correlated with the position of the −1 nucleosome (the first nucleosome peak upstream of the TSS) of each subset (−140 and −250 relative to the TSS; Fig. 2a). Both of the above analyses showed a positive correlation between GC content and nucleosome occupancy across the TSS. Next we sorted all PCG based on their GC content across the TSS, divided them into five quintiles (1st with the lowest GC content and 5th with the highest), and plotted the corresponding MNase-seq densities (Fig. 2b). Interestingly, this analysis revealed a negative correlation between GC content and nucleosome occupancy across the TSS. To uncouple the contribution of GC content from the TSS upstream and downstream regions, we generated another two sets of quintiles sorted based on their GC content either 1000 bp upstream or downstream of the TSS. Both analyses yielded a similar negative correlation: genes with high GC content either upstream or downstream of the TSS had lower nucleosome occupancy in the corresponding region (Fig. 2c and d). In addition, gene quintiles with an average of 48% (2nd quintile) to 53% (3rd quintile) GC content downstream of the TSS have the best nucleosome phasing (Fig. 2d). This may reflect stronger periodicity of SS (G/C) and WW (A/T) dinucleotides within the region, which would favor well-positioned nucleosomes [15] and would yield roughly equal GC and AT content overall. Together these results reveal that the correlation between GC content and nucleosome occupancy in rice is complex, possibly due to GC content acting as a *cis*-determinant, and also reflecting the involvement of other determinants, such as gene expression.

To elucidate the relationship between nucleosome patterns and gene expression in rice we carried out RNA sequencing (RNA-seq) analysis of the same tissues used for the MNase-seq experiments. Four RNA-seq libraries were generated consisting of two biological replicates for control (C1 and C2) and −Pi (P1 and P2). We obtained on average 97 million uniquely mapped reads for each library. To assess the reproducibility of mapped RNA-seq reads, we calculated Pearson’s correlation coefficient (PCC) of sequencing read abundance at each genomic position between C1 and C2 (PCC = 0.91) and P1 and P2 (PCC = 0.97) (Additional file 1: Fig. S1B). Biological replicates from RNA sequencing were kept separated for data analysis. Using the control samples, PCG were separated into five groups according to their expression levels (1st quintile highest and 5th quintile lowest) as determined by their FPKM values [43]. The MNase-seq densities of genes grouped by their expression were plotted at the window of ±1000 bp TSS (Additional file 3: Fig. S3A and B). We found that highly expressed genes had wider NDRs upstream of the TSS and had relatively lower nucleosome occupancy across the TSS than lower expressed genes. Moreover, evenly-spaced nucleosome arrays were more evident in highly expressed genes. These observations are consistent with previous studies on rice and Arabidopsis [7, 8, 13], and demonstrate that transcription is a strong determinant of nucleosome patterning in rice.

### Rice nucleosome patterns are linked to gene function

**Fig. 3:**
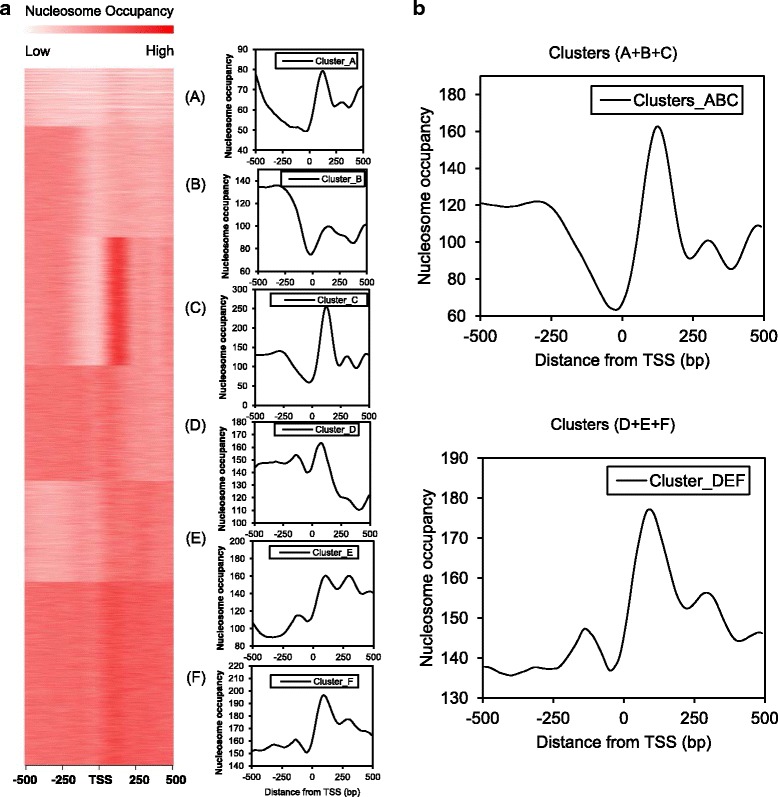
*k*-means clustering on nucleosome patterns across the TSS of rice genes. (**a**) *k*-means clustering of nucleosome patterns ±500 bp TSS of rice genes under control conditions. Right, individual heatmap profiles for gene clusters A − F at the TSS. (**b**) Average profiles for gene clusters ABC (type I gene) and DEF (type II gene) at the TSS

Experiments described above indicate a contribution of both *cis*- and *trans*-determinants to nucleosome patterning in rice. To further explore distinct nucleosome patterns across the TSS and their possible correlation with gene function, we performed *k*-means clustering of MNase-seq profiles ±500 bp TSS of PCG. Six clusters (A through F) of genes with distinct nucleosome patterns across the TSS were evident (Fig. 3A). GO term enrichment analysis [42] revealed that each of the six clusters had enriched GO terms (Additional file 4: Dataset 1). Interestingly, the clusters fell into two contrasting groups based on shared similar GO terms. Genes of clusters A, B, and C (type I, *n* = 15,400) were enriched in GO terms related to key biological processes, whereas clusters D, E, and F (type II, *n* = 20,700) were enriched in stress-related GO terms (Additional file 5: Fig. S4 and Additional file 4: Data S1). Hence, we termed type I genes “housekeeping” and type II genes “stress-responsive”. Consistent with a housekeeping role, type I genes had on average significantly higher expression levels than type II genes under control conditions (type I average FPKM = 22.47, type II average FPKM = 12.71, *p* < 2.2 × 10^−16^, Mann-Whitney *U* test). In addition, comparison of our type I housekeeping gene group with a housekeeping gene list identified by a previous study [44] based on consistent expression in multiple cell or tissue types, found that approximately 80% (3279/4243) of these previously reported housekeeping genes were included in our type I gene group.

Because studies have shown that the TATA box in the promoter region is associated with stress-responsive genes in yeast [45], we tested whether our type II stress-responsive gene group was enriched with genes containing a TATA box. We searched promoters of rice genes for the TATA consensus sequence (CTATAWAWA) previously reported [46]. Indeed, we found that type II genes were more likely to contain a TATA box within 50 bp upstream of the TSS than type I genes (2.6 folds, *p* = 6.90 × 10^−13^, Fisher’s exact test). Moreover, an average profile on type II genes across the TSS showed an evident −1 nucleosome where an NDR was found in type I genes (Fig. 3b), and genes with a TATA box within 50 bp upstream of the TSS (*n* = 1093) had a − 1 nucleosome at the same region (Fig. 6a). Together these results indicate that nucleosome patterns across the TSS are suggestive of gene function.

### Pi starvation induces large-scale nucleosome dynamics

To assess the impact of environmental perturbation on nucleosome patterns, we compared the nucleosome profiles identified in control shoot tissues with those in shoots harvested from plants subjected to a 24-h Pi starvation treatment. We measured total phosphorus and inorganic phosphate concentrations in rice seedlings from control and –Pi treatments prior to nucleosome and transcription profiling. Shoot P concentration significantly decreased (*p* < 0.01, *t*-test) while the root P concentration remained similar to that of the control (*p* > 0.05, *t*-test) after 24 h of Pi starvation (Additional file 6: Fig. S6A and B). In contrast, Pi concentration in shoots was unchanged (*p* > 0.05, *t*-test) while the root Pi concentration decreased (*p* < 0.05, *t*-test) after 24 h of Pi starvation (Additional file 6: Fig. S6C and D). These changes in Pi concentrations after 24 h of Pi starvation agree with a previous study which reflects the initiation of Pi starvation in rice seedlings [30].

**Fig. 4:**
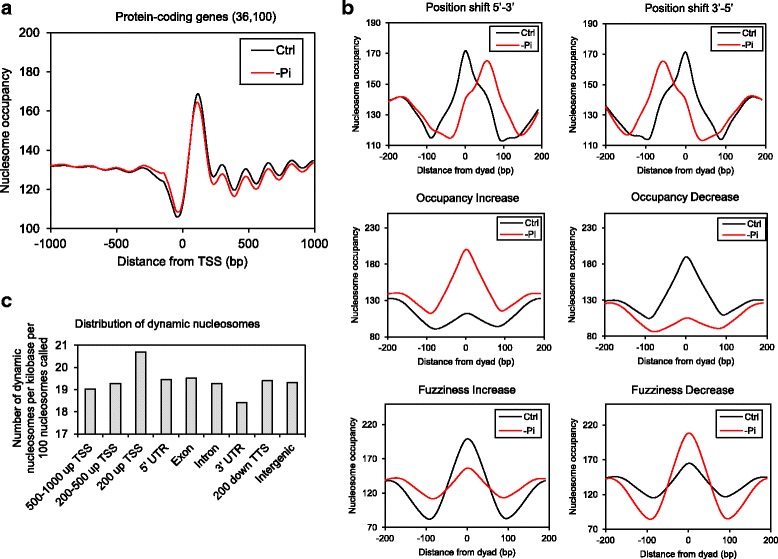
Changes in nucleosome patterns in response to phosphate starvation. (**a**) MNase-seq density across the TSS from 24-h control and −Pi rice shoot tissues. (**b**) Three types of nucleosome dynamics identified by DANPOS: position shift (range: 50–95 bp), occupancy change (FDR < 0.05) and fuzziness change (FDR < 0.05). Each averaged MNase-seq density graph is centered at the dyad of nucleosome that is found to house the change. (**c**) Number of different types of dynamic nucleosomes per kilo base (kb) per 100 nucleosomes called by DANPOS at the promoter (500–1000 bp upstream of the TSS, 200–500 bp upstream of the TSS and 200 bp upstream of the TSS), intragenic (5’UTR, exons, introns and 3’UTR), 200 bp downstream of the TTS, and intergenic regions

To investigate the impact of Pi starvation on genome-wide nucleosome patterns, we compared MNase-seq results from control and –Pi samples. We first examined nucleosome patterns across the TSS of genes. We found that nucleosome phasing remained largely the same between the control and –Pi samples, whereas –Pi samples had higher nucleosome occupancy 1000 bp upstream of the TSS (*p* < 2.2 × 10^−16^, Wilcoxon signed-rank test) but lower nucleosome occupancy 1000 bp downstream of the TSS (*p* < 2.2 × 10^−16^, Wilcoxon signed-rank test) as compared to control samples (Fig. 4A, Additional file 2: Fig. S2D). This raised the question of whether Pi starvation decreased nucleosome occupancy in coding regions but increased nucleosome occupancy in non-coding regions. To address this, we examined nucleosome occupancy changes in exons of genes under Pi starvation. Since longer exons allow the occupancy of more nucleosomes, we separated exons according to length: 170–240 bp, 315–350 bp, 480–550 bp, and 645–715 bp, which allows for the occupancy of one, two, three or four nucleosomes, respectively [37]. Both the control and –Pi samples showed strong nucleosome peaks and NDRs that marked intron-exon and exon-intron junctions (Additional file 7: Fig. S5). However, nucleosome occupancy was lower in exons and greater in introns of –Pi samples relative to the control samples (Additional file 7: Fig. S5), further supporting a major “redistribution” of nucleosomes from coding regions to non-coding regions in response to Pi starvation. A notable exception was at the TTS of PCG, at which the –Pi samples contained greater nucleosome occupancy relative to the controls (Additional file 2: Fig. S2C).

To better illustrate nucleosome dynamics in response to Pi starvation, we employed DANPOS, which defines accurate nucleosome maps and detects dynamic nucleosomes between samples [47]. This analysis revealed a substantial impact of Pi starvation on nucleosome occupancy and positioning. Using the nucleosome profile from control samples as a baseline, DANPOS identified 313,769 dynamic nucleosomes with either a position shift (range: 50–95 bp), occupancy change (FDR < 0.05), or fuzziness change (FDR < 0.05) associated with Pi starvation from two biological replicates (Fig. 4a). We analyzed the locations of the dynamic nucleosomes in the rice genome and found they were widely distributed in gene-related regions (1000 bp upstream of the TSS to 200 bp downstream of the TTS), and a large number of dynamic nucleosomes were mapped within 200 bp upstream of the TSS, 5’-UTR and exons (Fig. 4c).

### Nucleosome dynamics induced by Pi-starvation are enriched at differentially expressed genes

Our RNA sequencing libraries were prepared from total RNA depleted of ribosomal RNA, and quantification and comparison of intronic reads enabled us to more accurately capture changes in transcriptional activities than steady-state transcript levels inferred from conventional RNA-seq. We employed the iRNA-seq pipeline, which was demonstrated to perform at comparable qualities as global run-on (GRO)-seq and RNA polymerase II (RNAP II) ChIP-seq in determining genome-wide changes in transcriptional activities [48]. Using RNA-seq libraries from two biological replicates of control and –Pi samples, the iRNA-seq pipeline identified 134 up-regulated and 691 down-regulated genes (*padj* < 0.05) in response to 24-h Pi starvation in rice (Additional file 8: Dataset 2).

**Fig. 5:**
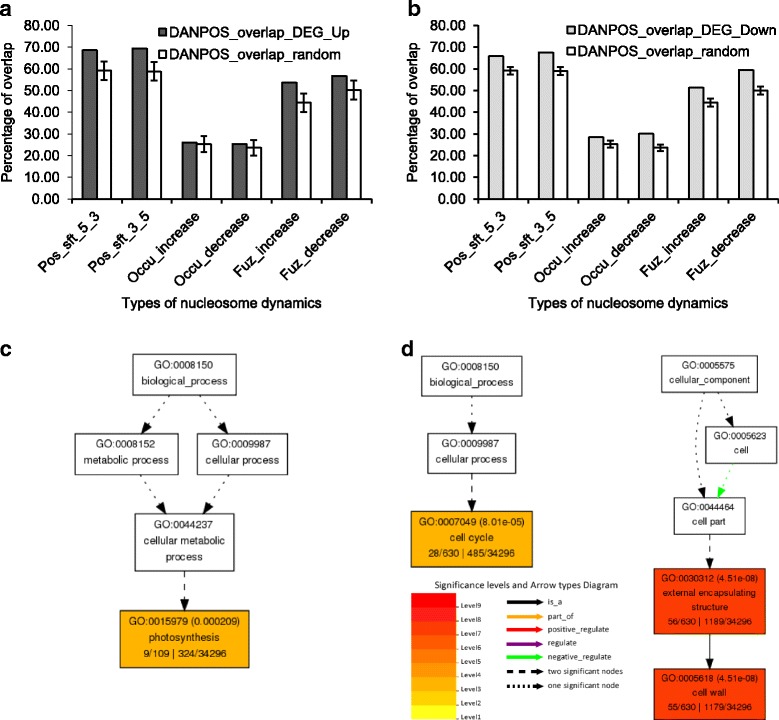
Changes in nucleosome patterns correlates with changes in transcription activities in response to phosphate starvation. (**a**) Percentage of 134 up-regulated genes (DEG_Up) called by iRNA-seq associated with dynamic nucleosomes called by DANPOS. Randomly selected (10,000 times) genes with the same size of DEGs were used as a control, and values are mean ± standard deviation. Pos_sft_5_3: 5′-3′ position shift; Pos_sft_3_5: 3′-5′ position shift; Occu_increase: increase in nucleosome occupancy; Occu_decrease: decrease in nucleosome occupancy; Fuz_increase: increase in nucleosome fuzziness; Fuz_decrease: decrease in nucleosome fuzziness. (**b**) Same analysis as (**a**) with 691 down-regulated genes (DEG_Dn). (**c**) AgriGO representation of the overrepresented Gene Ontology (GO) terms in the 130 genes up-regulated by 24 h of Pi starvation in the shoots via iRNA-seq associated with dynamic nucleosomes. Number in parenthesis represents the FDR value. (**d**) Same analysis as (**c**) with 678 down-regulated genes associated with dynamic nucleosomes

To investigate the correlation between transcriptional changes and nucleosome dynamics, we searched for differentially expressed (either up- or down-regulated) genes (DEGs) in which dynamic nucleosomes were present. We found that approximately 60%, 20%, and 50% of the DEGs were associated with nucleosome position shift, occupancy change, and fuzziness change respectively, within gene-related regions. These observed proportions were significantly higher than the same number of randomly selected genes as DEGs (*p* < 2.2 × 10^−16^, binomial test, with 10,000 iterations, Fig. 5A and B). Altogether, 130 out of 134 up-regulated genes and 678 out of 691 down-regulated genes (Additional file 8: Dataset 2) were found to contain dynamic nucleosomes, suggesting a strong correlation between transcriptional changes and nucleosome dynamics in response to Pi starvation in rice.

To better understand the roles of nucleosome dynamics and transcriptional changes in rice PSR, we sought functional information for the genes that exhibited changes in expression and nucleosome dynamics in response to Pi starvation. We first examined whether these genes were biased for type I (housekeeping) or type II (stress-responsive) genes. We found that both DEGs and genes with dynamic nucleosomes had higher overlaps with type II genes than type I genes (1.9 folds, *p* = 1.73 × 10^−3^ and 1.8 folds, *p* = 2.19 × 10^−3^ respectively, Fisher’s exact test). Next we carried out GO term enrichment analysis on DEGs associated with dynamic nucleosomes. Up-regulated genes with nucleosome dynamics were involved in photosynthesis (GO:0015979, FDR = 2.09 × 10^−4^) whereas down-regulated genes containing dynamic nucleosomes were enriched in GO terms including cell cycle (GO:0004079, FDR = 8.01 × 10^−5^) and cell wall (GO:0005618, FDR = 4.51 × 10^−8^) (Fig. 5C and D, Additional file 9: Dataset 3). These results support a modulation of photosynthesis and growth in rice shoots in response to Pi starvation through changes in gene expression that are linked to corresponding changes in nucleosome positioning and occupancy.

**Fig. 6:**
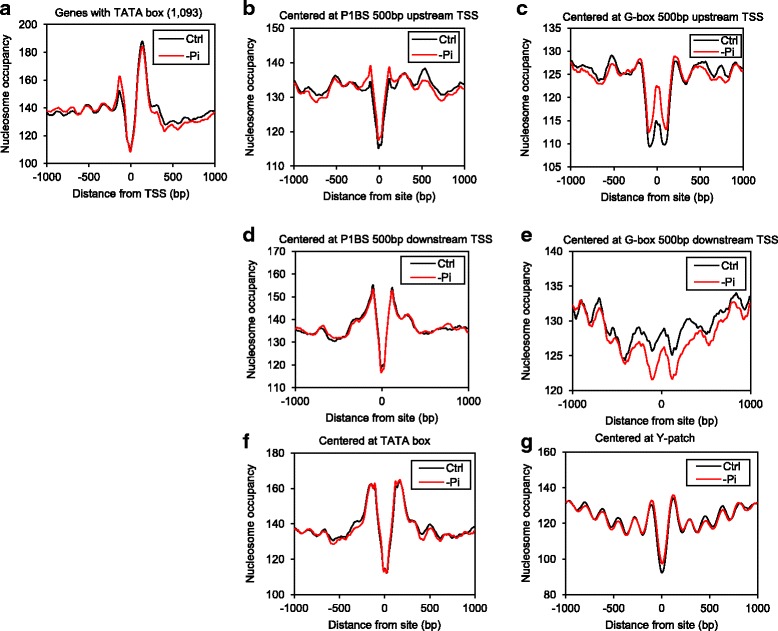
Changes in nucleosome occupancy at *cis*-elements in response to phosphate starvation. (**a**) MNase-seq density across the TSS of genes that contain TATA box under control (Ctrl) and −Pi conditions (*n* = 1093). MNase-seq density of (**b**) P1BS within 500 bp upstream of the TSS; (**c**) G-box within 500 bp upstream of the TSS; (**d**) P1BS within 500 up downstream of the TSS; (**e**) G-box within 500 bp downstream of the TSS; (**f**) TATA box; (**g**) Y-patch. Plots are centered at the sequence of interest under control (Ctrl) and −Pi conditions

In yeast, nucleosome remodeling is necessary for full induction of several yeast PHO regulon genes, which are activated in response to Pi deficiency [18, 49]. For example, in the case of the yeast PHO5 acid phosphatase gene, a nucleosome blocks a promoter binding site of the Pho4 transcription activator under Pi-replete conditions, repressing PHO5 transcription. Upon Pi deficiency, nucleosome remodeling exposes the *cis*-element making it accessible to Pho4, which then initiates PHO5 transcription [18]. In rice, the OsPHR2 transcription factor induces expression of numerous Pi-related genes in response to Pi starvation by binding to the P1BS *cis*-element (consensus sequence GNATATNC [50]). To investigate the possible nucleosome dynamics at the P1BS element in response to 24-h of Pi starvation, we compared nucleosome occupancy between control and –Pi samples at occurrences of the P1BS motif found within 500 bp upstream of the TSS. Nucleosome occupancy was higher in the –Pi samples near the center of the P1BS motif (± 250 bp) as compared to the control samples (*p* = 1.58 × 10^−9^, Wilcoxon signed-rank test, Fig. 6B). To examine the specificity of nucleosome dynamics at the P1BS motif in response to Pi starvation, we also compared nucleosome occupancy centered at the G-box (CACGTG) found within 500 bp upstream of the TSS, which is a known *cis*-element that regulates jasmonic acid (JA)-responsive gene expression [15], and we also observed increased nucleosome occupancy at the G-box motif in the –Pi samples (*p* = 5.80 × 10^−10^, Wilcoxon signed-rank test, Fig. 6C). Moreover, nucleosome occupancy centered at both elements found within 500 bp downstream of the TSS were lower in the –Pi samples than the control samples (*p* ≤ 6.19 × 10^−9^, Wilcoxon signed-rank test, Fig. 6D and E). These results indicate that the nucleosome dynamics we observed at the P1BS motif may not be unique to Pi starvation but rather were the results of a broad effect of higher nucleosome occupancy upstream of the TSS and lower nucleosome occupancy downstream of the TSS caused by Pi starvation (Fig. 4a and Additional file 2: Fig. S2D). We further plotted MNase-seq density of regions centered at TATA box and Y-patch (consensus sequence CYTCYYCCYC), a core promoter element in rice genes [46], and found all TF binding sites including P1BS were depleted of nucleosomes compared with surrounding regions while strong nucleosome peaks were found on the boundaries of binding sites regardless of Pi starvation (Fig. 6b, f, and g).

## Discussion

In this study, we determined nucleosome patterns in rice genes and explored the correlations among nucleosome patterns, gene structure, gene function, and transcription activities. We also captured nucleosome dynamics along with transcriptional changes in response to 24 h of Pi starvation and showed a significant relationship between the two.

We found higher nucleosome occupancy surrounding the TSS and the TTS compared to the gene body suggesting the role of chromatin organization in defining the initiation and termination of transcription (Fig. 1a, Additional file 2: Fig. S2A and B). The presence of evenly-spaced nucleosome arrays downstream of the TSS and NDRs immediately upstream of the TSS at PCG further indicates the impact of chromatin organization on transcriptional activities (Fig. 1a). The nucleosome patterns we found in rice genes were largely consistent with patterns in yeast and human [6, 12], but as with Arabidopsis, rice genes lacked evenly-spaced nucleosome arrays upstream of the TSS (Fig. 1a). Studies on yeast and human used single cell types but studies on Arabidopsis and rice, including this study, used homogenized plant tissues consisting of multiple cell types that could contribute to the heterogeneity of nucleosome patterns in the promoter region due to tissue-specific expression differences. However, a genome-wide study on the single-celled protozoan *Tetrahymena thermophila* also showed nucleosome phasing downstream but not upstream of the TSS [40]. This result is possibly due to cell-to-cell differences within the same *T. thermophilia* culture, since studies on single-cell nucleosome mapping in yeast showed that different cells in the same yeast culture possessed different nucleosome patterns in the promoter region [51]. Another explanation is that that since the plant and *T. thermophila* genomes contain larger numbers of genes compared to yeast and human, they may contain greater variability in nucleosome patterns at the promoter region which interferes with the detection of nucleosome arrays. The variable length of DHS was shown to mask the detection of nucleosome arrays upstream of the TSS in rice [13]. We identified two subsets of genes that had evenly-spaced nucleosome arrays upstream of the TSS and the nucleosome arrays were canceled out while an average profile was plotted suggesting nucleosome patterns at individual genes may differ from stereotypical averaged profiles (Fig. 1B). Nonetheless, the arrays downstream of the TSS were “in phase” in both subsets of genes indicating the role of coding sequence characteristics and transcription activities in establishing nucleosome arrays.

Positions of nucleosomes in the genome are not random but rather are controlled by the combination of both *cis-* and *trans-*determinants. We observed lower nucleosome occupancy at relatively highly expressed genes, and higher expressed genes had larger distance between the TSS and the +1 nucleosome (the first nucleosome peak downstream of the TSS) with a wider 5’ NDR (Additional file 3: Fig. S3). These observations agree with previous findings in Arabidopsis and rice, reflecting the correlation between open chromatin architecture and transcription [7, 8, 13]. It has been widely reported that GC- and AT-rich sequences favor and disfavor nucleosome formation, respectively [11]. Indeed, enriched SS (G/C) dinucleotides in the cores of well-positioned nucleosomes and enriched WW (A/T) dinucleotides in nucleosome flanking sequences has been observed for yeast [9], Drosophila [14], human [12], rice [15], and *T. thermophila* [40]. However, seemingly contradictory correlations among GC content and nucleosome occupancy have been observed. For example, the GC content of randomly selected genomic fragments of yeast and human are positively correlated with nucleosome occupancy, whereas a negative correlation is observed for rice and Arabidopsis [8, 52]. Also, GC content has a negative correlation with nucleosome occupancy at predicted transcription factor binding sites in human and rice [8, 52]. Herein, we compared the relationship between nucleosome occupancy and GC content in several ways. First we mapped GC content distribution across the TSS, which correlated with nucleosome occupancy on a “macro” level: relatively low levels upstream of the TSS and higher levels downstream (Fig. 2A). By comparing the −140 and −250 gene subsets, we also showed a positive correlation on a “micro” level: peaks of GC% overlapped with the positions of the corresponding −1 nucleosome peaks (Fig. 2a). These observations are consistent with GC-rich sequences favoring nucleosome occupancy. However, grouping PCG into GC quintiles showed an obvious negative correlation across the TSS, particularly in the downstream region (Fig. 2d). Together these results support the hypothesis that GC content intrinsically influences nucleosome occupancy, but that other determinants including transcription contribute to nucleosome occupancy. We also observed that genes with on average 48% to 53% of GC content downstream of the TSS have better nucleosome phasing, suggesting the influence of GC content on nucleosome phasing downstream of the TSS (Fig. 2d). Future studies on DNA sequence arrangement at positioned nucleosomes within different regions of rice genes may reveal how DNA sequence (content and arrangement) affects genome-wide nucleosome positioning.

Distinct nucleosome patterns across the TSS of rice genes make it possible to categorize genes based on their nucleosome patterns across the TSS. We show two groups of rice genes (type I and type II) clustered by their distinct nucleosome patterns across the TSS with distinct gene functions (Fig. 3). Type I genes have wide 5’ NDRs correlating with relatively high transcription rate as housekeeping genes, whereas type II stress-responsive genes have nucleosomes positioned on either side of the TSS which may create obstacles for transcription machinery as well as serving as the landmarks for TF and chromatin remodelers to recognize under induced conditions. Nucleosome patterns show strong correlations with the above-discussed gene characteristics, suggesting the possibility of inferring such characteristics (e.g. expression and function) from the associated nucleosome patterns. Indeed, recent studies have shown promising results on predicting tumor gene expression and tissue of origin from nucleosome patterns of cell-free DNA in human plasma [53, 54]. Together, our findings support a conserved correlation between nucleosome patterns and gene characteristics in eukaryotes, which could benefit the agricultural and medical communities in the near future.

The second part of our work revealed changes in nucleosome patterns and transcription activities in response to 24 h of Pi starvation and their correlations in rice. Genome-wide studies in yeast showed dynamic relationships among nucleosome patterns, gene expression and TF binding responding to heat shock and oxidative stress [19, 20]. In Arabidopsis, 1-h of coronatine (COR) treatment was shown to trigger changes in transcript abundance, but differential gene expression changes were not correlated with nucleosome occupancy changes in genes [8]. Our data show that 24 h of Pi starvation induces genome-wide nucleosome reorganization, especially at the regions surrounding the TSS and the coding sequence (Fig. 4 and Additional file 7: Fig. S5), and we found a strong correlation between nucleosome dynamics at genes and transcriptional changes (Fig. 5a and b). Among the three types of nucleosome dynamics (position shift, occupancy change and fuzziness change), we found that position shift and fuzziness are more relevant to DEGs by Pi starvation than occupancy change (Fig. 5a and b), indicating specificity of nucleosome dynamics and its association with biological functions, as demonstrated in yeast previously [47]. Histone variants H2A.Z and H3.3, and acetylated and methylated histones are usually enriched in the +1 nucleosome and contribute to the flexibility of nucleosome occupancy which assists nucleosome eviction and assembly of the pre-initiation complex [55]. H2A.Z deposition at PSR genes was shown to be correlated with Pi starvation responses in Arabidopsis [36]. Our observation of enriched nucleosome dynamics across the TSS during Pi starvation may reflect the replacement of canonical histones with variants such as H2A.Z as well as post-translational modifications of histones at the TSS (Fig. 4A and C).

Unlike previous studies in yeast and Arabidopsis, we employed iRNA-seq on ribosome-depleted total RNA to capture changes in nascent RNA transcript instead of quantification of steady-state RNA transcript in response to an environmental perturbation, improving the identification of DEGs that are specific to the treatment. With 24 h of Pi starvation, photosynthesis was up-regulated while cell cycle and cell wall synthesis were down-regulated (Fig. 5c and d). These observations are consistent with a recent study on Pi starvation in the dinoflagellate *Amphidinium carterae*, which showed decreased cell division in response to Pi starvation, but continued photosynthetic capability [24]. We propose a similar adaptation strategy carried out by rice in response to short-to-medium term Pi starvation, which includes enhancing photosynthesis to accumulate energy, and reducing DNA replication, cell division, and cell wall expansion to minimize Pi usage. In Arabidopsis, the DNA element G-box plays a role in regulating JA-responsive gene expression, and it was shown that nucleosomes were depleted at this element regardless of COR treatment, which mimics JA responses [8]. We show similar patterns at the rice core promoter elements TATA box and Y-patch, as well as P1BS; that nucleosomes were depleted at those elements regardless of Pi starvation (Fig. 6b, f, and g). This may result from constant binding of *trans*-acting factors to assist a rapid transcriptional change in response to stress.

We observed a significant drop in Pi concentration after 24 h of Pi starvation in the roots (Additional file 6: Fig. S6D), and future nucleosome and transcription profiling studies could include roots where Pi is sensed and acquired. We anticipate that monitoring nucleosome dynamics with transcriptional changes at multiple time points of Pi starvation with Pi re-supply in a single cell of multiple plant cell types, and identification of PSR-related chromatin remodelers could broaden the understanding on PSR in rice and eventually provide useful resources for the agricultural community.

## Conclusions

Our work provides a high-resolution map of nucleosomes in the rice genome, along with its transcriptional profile in response to Pi starvation. We also provide measurements on the correlation between nucleosome dynamics and transcriptional changes. We demonstrate how gene characteristics may affect nucleosome patterns in rice genes. Our findings advance the understanding towards eukaryotic gene regulation at the chromatin level.

## Methods

### Plant material and growth conditions

Rice (*Oryza sativa ssp. japonica* cv. Nipponbare) seeds were surface-sterilized with 5% bleach and rinsed, and soaked in sterile distilled water at 37 °C in the dark for 3 days for pre-germination. Seeds were allowed to germinate at 22 °C under a 16 h/8 h day/night cycle for 14 days. Rice seedlings were transferred to half-strength Hoagland’s nutrient media (2.5 mM KNO_3_, 1 mM KH_2_PO_4_, 3.5 mM Ca(NO_3_)_2_·4H_2_O, 1 mM MgSO_4_·7H_2_O, 5 μM MnCl_2_·4H_2_O, 0.07 mM NaMoO_4_, 0.02 mM H_3_BO_3_, 0.3 μM ZnSO_4_·7H_2_O, 0.2 μM CuSO_4_·5H_2_O, 0.014 mM Fe(EDTA)) for 21 days in a growth room under non-sterile conditions. For nutrient treatment, seedlings were transferred to fresh half-strength Hoagland’s or the same media lacking phosphorus (KH_2_PO_4_) for 24 h. The hydroponic experiments were performed under 16 h/8 h day/night cycles and temperature was kept at 22 °C in a growth room. The pH of the solution was adjusted to 5.5 and the solution was renewed every 7 days. Shoots (green tissues) and roots from the seedlings were flash frozen in liquid nitrogen, and stored at −80 °C.

### MNase-seq

The EZ Nucleosomal DNA Prep Kit (Zymo Research, Irvine, CA) was used for nuclei isolation and mono-nucleosomal DNA preparation from plant tissues. The nuclei isolation and MNase treatment were performed according to the manual with modifications. Briefly, tissues were ground in liquid nitrogen and re-suspended in HBM buffer (25 mM Tris pH 7.6, 0.44 M sucrose, 10 mM MgCl_2_, 0.1% Triton X, 2 M spermidine, and 10 mM β-mercaptoethanol). The mixture was filtered with miracloth and centrifuged for 60 min at 2000×*g*. Isolated nuclei were washed with Nuclei Prep Buffer (Zymo Research). The prepared nuclei were treated with MN Digestion buffer (Zymo research). The nuclei were digested with 1 U (final concentration of 0.004 U/μl) micrococcal nuclease (MNase, Zymo Research) for 30 min at room temperature. The digestion was stopped by addition of 5× MN Stop Buffer (Zymo Research). Nucleosomal DNA was purified by addition of DNA Binding Buffer (Zymo Research), and centrifuged with a Zymo-spin IIC Column in a Collection Tube (Zymo Research). DNA was washed with DNA Wash Buffer and eluted with warm DNA Elution Buffer (Zymo Resarch). Purified DNA was run on a 2% agarose gel containing ethidium bromide and visualized under UV light. The mono-nucleosomal DNA (~150 bp band) was excised from the gel and purified with a gel purification kit (Qiagen, Hilden, Germany).

Approximately 500 ng of MNase-digested mono-nucleosomal DNA from each sample was used for Illumina library generation. Library construction and deep sequencing were performed by the Roy J. Carver Biotechnology Center at the University of Illinois at Urbana-Champaign using an Illumina HiSeq 2000 platform (Illumina, San Diego, CA). Raw data comprised 100 bp of single-ended reads. Illumina sequencing reads were mapped to the rice genome (MSU7) [39] using Bowtie (version 1.1.2) [56] and only uniquely mapped reads were considered for further analysis. Approximately 63 million reads per sample (~15× coverage) were obtained. Correlations among mapped sequencing samples were analyzed using DeepTools [57]. Mapped reads were subject to GC content bias correction as previously described [57, 58]. Nucleosome positions were identified and analyzed using the dpos function of DANPOS software with default settings for two sets of biological replicates (control and –Pi) [47]. An FDR of 0.05 was used for dynamic nucleosome calling, except for position shift where a range setting of 50–95 bp was used. Genome-wide nucleosome patterns were plotted using DANPOS [47] or ngs.plot [59]. Nucleosome occupancy were determined as averaged reads per million mapped reads. IGV was used to visualize mapped reads to the reference genome [60]. The genome annotation was obtained from MSU Rice Genome Annotation Project website (http://rice.plantbiology.msu.edu/) and parsing of dynamic nucleosome locations were performed using BedTools (version 2.26.0) [61].

### RNA-seq

The total RNA from plant tissues was extracted using an RNeasy Plant Mini Kit (Qiagen) according to the manufacturer’s instructions. On-column DNase digestion was performed on the extracted total RNA with an RNase-free DNase set (Qiagen) according to manufacturer’s instructions to reduce DNA contamination. Approximately 2 μg of DNase-treated total RNA from each sample was used for sequencing library construction. For sequencing library preparation, ribosomal RNA was removed with Ribo-Zero™ rRNA Removal Kit (Plant) and the remaining RNA was processed with the TruSeq Stranded mRNA library construction kit (Illumina) starting at the fragment/elute step (no mRNA selection). Library construction and deep sequencing were performed by the Roy J. Carver Biotechnology Center at the University of Illinois at Urbana-Champaign using an Illumina HiSeq 2500 platform (Illumina). Raw data comprised 100 bp of single-ended reads.

Ribosomal RNA reads were further removed by mapping sequencing reads to all known rice ribosomal DNA sequences obtained from the *Oryza* repeat database (http://plantrepeats.plantbiology.msu.edu/index.html) using Bowtie [56] with default settings, and reads that failed to align were kept. The remaining reads were then mapped to the rice genome (MSU7) [39] using TopHat2 [62] with the following settings: --b2-sensitive -g 1, allowing only one hit for each read. Approximately 97 million reads per sample were obtained. Correlations among mapped sequencing samples were analyzed using DeepTools [57]. Cufflinks [43] was used to determine transcript abundance (FPKM) of each gene from two control replicates. Differential expression of biological replicates between control and –Pi were determined by the iRNAseq pipeline [48] based on the sequencing reads abundance at the introns with default settings. Records from the output file ‘introns.txt’ of the iRNA-seq pipeline was filtered for adjusted *p*-value (Padj) < 0.05, and then genes with positive log2_FC were determined as up-regulated, and genes with negative values were determined as down-regulated. GO term enrichment analysis were performed using AgriGO with default settings [42].

### Statistical methods

Statistical methods were used as previously reported: We used *k*-means clustering as the clustering method to classify genes based on nucleosome patterns across the TSS [59]. We used Mann-Whitney *U* test to address the FPKM differences between two groups of genes with different sizes [8, 15, 54], and Fisher’s exact test to address the overrepresentations of genes [8, 30, 48], and Wilcoxon signed-rank test to address differences in nucleosome occupancy [19, 48], and binomial test to address the bias over random [13, 63], and Student’s *t*-test to address differences in P and Pi measurements from plant tissues [31, 33]. All statistical tests were performed using R (v 3.2.3, https://www.r-project.org/) and significance was defined as *p* < 0.05.

### Quantification of total phosphorus (P) and inorganic phosphate (Pi)

Plant tissues were rinsed thoroughly in distilled water before analyses. Quantification of total phosphorus was conducted by an acid digestion method as described previously [64]. Briefly, 0.5 g of the dried leaf and root tissues were digested with 5.0 mL of concentrated HNO_3_ in a heat block at 125 °C for 2.5 h followed by repeated addition of 3 mL 30% H_2_O_2_ until the digest was clear. The temperature of the heat block was reduced to 80 °C for the residue to dry. Colorless dry residue was dissolved in 20 mL deionized water and analyzed by inductively coupled plasma emission spectroscopy (ICP) using (NH_4_)_2_HPO4 as the standard in the LSU Soil Testing & Plant Analysis Laboratory.

Quantification of inorganic phosphate (Pi) was conducted by grinding plant tissue in liquid nitrogen and dissolving in distilled water. Pi was quantified by the molybdate assay [65], and a standard curve was generated using KH_2_PO_4_.

## Additional files

Additional file 1: **Figure S1.** Reproducibility of MNase-seq and RNA-seq. C1, control replicate 1; C2, control replicate 2; P1, −Pi replicate 1, P2, −Pi replicate 2. (A) Clustered heatmap of mapped MNase-seq samples with Spearman correlation coefficient (*ρ*). The distances among sample pairs are determined as 1-*ρ*. (B) Clustered heatmap of mapped rmRNA-seq samples with Pearson correlation coefficient (*r*). The distances among sample pairs are determined as 1-*r*. (PDF 161 kb)

Additional file 2: **Figure S2.** Nucleosome patterns across the transcription termination site and gene body of rice genes. Regions 1000 bp upstream and downstream of the transcription termination site (TTS or 3′ boundary of the element), and gene body (GB, from TSS (5′ boundary) to TTS (3′ boundary) of a gene) were used to plot MNase-seq density under control conditions (A and B) and control and –Pi (C and D). (A) MNase-seq density for all rice genes across the TTS under control conditions. (B) MNase-seq density for all rice genes across the GB under control conditions. (C) MNase-seq density of PCG across the TTS from 24-h control and –Pi rice shoots. (D) MNase-seq density of PCG across the GB from 24-h control and –Pi rice shoots. (PDF 77 kb)

Additional file 3: **Figure S3.** Correlations between nucleosome patterns and gene expression. (A) Heatmap of MNase-seq density of PCG sorted by their expression level under control conditions from RNA-seq analysis of the same tissue (1st highest, 5th lowest). The vertical line in the middle of the heatmap indicates the TSS. (B) Average plot of the same data with genes grouped by their expression levels. (PDF 65 kb)

Additional file 4: Dataset 1. GO term enrichment for six clusters of genes. (ZIP 1029 kb)

Additional file 5: **Figure S4.** Significantly enriched GO terms for clusters ABC (type I gene) and DEF (type II gene). The color of the node represents the corrected *p*-value with a color scale ranging from yellow (corrected *p*-value = 0.05) to dark orange (corrected *p*-value = 5 × 10^−7^). (PDF 443 kb)

Additional file 6: **Figure S6.** Changes of total phosphorus and inorganic phosphate concentrations in response to phosphate starvation. All values are the mean ± standard error of the mean; *n* = 3 biological replicates with 3 technical repeats each. DW, dry weight; FW, fresh weight. (A) Total phosphorus (P) concentrations for shoots of 5-week-old seedlings grown under full nutrient (Ctrl) and Pi-starvation (−Pi) conditions. (B) Total P concentrations for roots of 5-week-old seedlings grown under full nutrient (Ctrl) and Pi-starvation (−Pi) conditions. (C) Inorganic phosphate (Pi) concentrations for shoots of 5-week-old seedlings grown under full nutrient (Ctrl) and Pi-starvation (−Pi) conditions. (D) Pi concentrations for roots of 5-week-old seedlings grown under full nutrient (Ctrl) and Pi-starvation (−Pi) conditions. (PDF 38 kb)

Additional file 7: **Figure S5.** Changes in nucleosome occupancy at the exons of rice genes in response to phosphate starvation. MNase-seq density of exons grouped according to their length: (A) 170–240 bp; (B) 315–350 bp; (C) 480–550 bp (D) 645–715 bp under control and −Pi conditions. Plots are centered at the 5′ boundaries of the exons. (PDF 58 kb)

Additional file 8: Dataset 2. Gene lists for 134 up-regulated genes, 130 up-regulated genes with dynamic nucleosomes, 691 down-regulated genes, and 678 down-regulated genes with dynamic nucleosomes. (XLSX 22 kb)

Additional file 9: Dataset 3. GO term enrichment for up- and down-regulated genes associated with dynamic nucleosomes. (ZIP 20 kb)

## Abbreviations

ChIP: chromatin immunoprecipitation
COR: coronatine
DEG: differentially expressed gene
DHS: DNase I hypersensitivity site
FDR: false discovery rate
FPKM: fragments per kilobase of transcript per million mapped reads
GB: gene body
GO: gene ontology
MNase-seq: sequencing of micrococcal nuclease digested chromatin
NDR: nucleosome depleted region
NFR: nucleosome free region
PCC: Pearson’s correlation coefficient
PCG: protein-coding gene
PSG: pseudogenes
PSR: phosphate starvation response
RNAP II: RNA polymerase II
RNA-seq: RNA sequencing
SCC: Spearman’s correlation coefficient
TE: transposable element
TEG: transposable element related gene
TSS: transcription start site
TTS: transcription termination site

## References

[1] Luger K, Mader AW, Richmond RK, Sargent DF, Richmond TJ. Crystal structure of the nucleosome core particle at 2.8 Å resolution. Nature. 1997;389(6648):251–60. 10.1038/38444 9305837

[2] Annunziato AT (2005). Split decision: what happens to nucleosomes during DNA replication. J Biol Chem.

[3] Price BD, D'Andrea AD (2013). Chromatin remodeling at DNA double-strand breaks. Cell.

[4] Pulivarthy SR, Lion M, Kuzu G, Matthews AG, Borowsky ML, Morris J, Kingston RE, Dennis JH, Tolstorukov MY, Oettinger MA (2016). Regulated large-scale nucleosome density patterns and precise nucleosome positioning correlate with V(D)J recombination. Proc Natl Acad Sci U S A.

[5] Radman-Livaja M, Rando OJ (2010). Nucleosome positioning: how is it established, and why does it matter. Dev Biol.

[6] Lee W, Tillo D, Bray N, Morse RH, Davis RW, Hughes TR, Nislow CA (2007). High-resolution atlas of nucleosome occupancy in yeast. Nat Genet.

[7] Li G, Liu S, Wang J, He J, Huang H, Zhang Y, Xu L. ISWI proteins participate in the genome-wide nucleosome distribution in Arabidopsis. Plant J. 2014;78(4):706–14. 10.1111/tpj.12499 24606212

[8] Liu MJ, Seddon AE, Tsai ZT, Major IT, Floer M, Howe GA, Shiu SH (2015). Determinants of nucleosome positioning and their influence on plant gene expression. Genome Res.

[9] Mavrich TN, Ioshikhes IP, Venters BJ, Jiang C, Tomsho LP, Qi J, Schuster SC, Albert I, Pugh BF. A barrier nucleosome model for statistical positioning of nucleosomes throughout the yeast genome. Genome Res. 2008;18(7):1073–83. 10.1101/gr.078261.108 PMC2493396 18550805

[10] Struhl K, Segal E (2013). Determinants of nucleosome positioning. Nat Struct Mol Biol.

[11] Tillo D, Hughes TR. G+C content dominates intrinsic nucleosome occupancy. BMC Bioinformatics. 2009;10(1):442. 10.1186/1471-2105-10-442 PMC2808325 20028554

[12] Valouev A, Johnson SM, Boyd SD, Smith CL, Fire AZ, Sidow A (2011). Determinants of nucleosome organization in primary human cells. Nature.

[13] YF W, Zhang WL, Jiang JM (2014). Genome-wide nucleosome positioning is orchestrated by genomic regions associated with DNase I hypersensitivity in rice. PLoS Genet.

[14] Mavrich TN, Jiang C, Ioshikhes IP, Li X, Venters BJ, Zanton SJ, Tomsho LP, Qi J, Glaser RL, Schuster SC, et al. Nucleosome organization in the Drosophila genome. Nature. 2008;453(7193):358–62. 10.1038/nature06929 PMC2735122 18408708

[15] Zhang T, Zhang W, Jiang J (2015). Genome-wide nucleosome occupancy and positioning and their impact on gene expression and evolution in plants. Plant Physiol.

[16] Secco D, Wang C, Shou H, Whelan J. Phosphate homeostasis in the yeast *Saccharomyces cerevisiae*, the key role of the SPX domain-containing proteins. FEBS Lett. 2012;586(4):289–95. 10.1016/j.febslet.2012.01.036 22285489

[17] Almer A, Rudolph H, Hinnen A, Horz W (1986). Removal of positioned nucleosomes from the yeast PHO5 promoter upon PHO5 induction releases additional upstream activating DNA elements. EMBO J.

[18] Barbaric S, Luckenbach T, Schmid A, Blaschke D, Horz W, Korber P (2007). Redundancy of chromatin remodeling pathways for the induction of the yeast PHO5 promoter in vivo. J Biol Chem.

[19] Huebert DJ, Kuan P-F, Keleş S, Gasch AP (2012). Dynamic changes in nucleosome occupancy are not predictive of gene expression dynamics but are linked to transcription and chromatin regulators. Mol Cell Biol.

[20] Shivaswamy S, Bhinge A, Zhao Y, Jones S, Hirst M, Iyer VR (2008). Dynamic remodeling of individual nucleosomes across a eukaryotic genome in response to transcriptional perturbation. PLoS Biol.

[21] Raghothama KG (1999). Phosphate acquisition. Annu Rev Plant Physiol Plant Mol Biol.

[22] Conley DJ, Paerl HW, Howarth RW, Boesch DF, Seitzinger SP, Havens KE, Lancelot C, Likens GE (2009). Ecology. Controlling eutrophication: nitrogen and phosphorus. Science.

[23] Elser J, Bennett E (2011). Phosphorus cycle: a broken biogeochemical cycle. Nature.

[24] Li M, Shi X, Guo C, Lin S. Phosphorus deficiency inhibits cell division but not growth in the dinoflagellate *Amphidinium carterae*. Front Microbiol. 2016;7(826) 10.3389/fmicb.2016.00826 PMC4887478 27313570

[25] Vance CP, Uhde-Stone C, Allan DL (2003). Phosphorus acquisition and use: critical adaptations by plants for securing a nonrenewable resource. New Phytol.

[26] Hu B, Chu C (2011). Phosphate starvation signaling in rice. Plant Signal Behav.

[27] Rouached H, Arpat AB, Poirier Y (2010). Regulation of phosphate starvation responses in plants: signaling players and cross-talks. Mol Plant.

[28] Wang Z, Ruan W, Shi J, Zhang L, Xiang D, Yang C, Li C, Wu Z, Liu Y, Yu Y (2014). Rice SPX1 and SPX2 inhibit phosphate starvation responses through interacting with PHR2 in a phosphate-dependent manner. Proc Natl Acad Sci U S A.

[29] Wu P, Shou H, Xu G, Lian X (2013). Improvement of phosphorus efficiency in rice on the basis of understanding phosphate signaling and homeostasis. Curr Opin Plant Biol.

[30] Secco D, Jabnoune M, Walker H, Shou H, Wu P, Poirier Y, Whelan J (2013). Spatio-temporal transcript profiling of rice roots and shoots in response to phosphate starvation and recovery. Plant Cell.

[31] Secco D, Wang C, Shou H, Schultz MD, Chiarenza S, Nussaume L, Ecker JR, Whelan J, Lister R. Stress induced gene expression drives transient DNA methylation changes at adjacent repetitive elements. elife. 2015;4 10.7554/eLife.09343 PMC4534844 26196146

[32] Wu P, Ma L, Hou X, Wang M, Wu Y, Liu F, Deng XW (2003). Phosphate starvation triggers distinct alterations of genome expression in Arabidopsis roots and leaves. Plant Physiol.

[33] Zheng L, Huang F, Narsai R, Wu J, Giraud E, He F, Cheng L, Wang F, Wu P, Whelan J (2009). Physiological and transcriptome analysis of iron and phosphorus interaction in rice seedlings. Plant Physiol.

[34] Iglesias J, Trigueros M, Rojas-Triana M, Fernández M, Albar JP, Bustos R, Paz-Ares J, Rubio V (2013). Proteomics identifies ubiquitin–proteasome targets and new roles for chromatin-remodeling in the Arabidopsis response to phosphate starvation. J Proteome.

[35] Kuo H-F, Chang T-Y, Chiang S-F, Wang W-D, Charng Y-Y, Chiou T-J (2014). Arabidopsis inositol pentakisphosphate 2-kinase, AtIPK1, is required for growth and modulates phosphate homeostasis at the transcriptional level. Plant J.

[36] Smith AP, Jain A, Deal RB, Nagarajan VK, Poling MD, Raghothama KG, Meagher RB (2010). Histone H2A.Z regulates the expression of several classes of phosphate starvation response genes but not as a transcriptional activator. Plant Physiol.

[37] Chodavarapu RK, Feng SH, Bernatavichute YV, Chen PY, Stroud H, YC Y, Hetzel JA, Kuo F, Kim J, Cokus SJ (2010). Relationship between nucleosome positioning and DNA methylation. Nature.

[38] Fincher JA, Vera DL, Hughes DD, McGinnis KM, Dennis JH, Bass HW (2013). Genome-wide prediction of nucleosome occupancy in maize reveals plant chromatin structural features at genes and other elements at multiple scales. Plant Physiol.

[39] Kawahara Y, de la Bastide M, Hamilton JP, Kanamori H, McCombie WR, Ouyang S, Schwartz DC, Tanaka T, Wu J, Zhou S (2013). Improvement of the *Oryza sativa* Nipponbare reference genome using next generation sequence and optical map data. Rice.

[40] Xiong J, Gao S, Dui W, Yang W, Chen X, Taverna SD, Pearlman RE, Ashlock W, Miao W, Liu Y (2016). Dissecting relative contributions of cis- and trans-determinants to nucleosome distribution by comparing Tetrahymena macronuclear and micronuclear chromatin. Nucleic Acids Res.

[41] Vera DL, Madzima TF, Labonne JD, Alam MP, Hoffman GG, Girimurugan SB, Zhang J, McGinnis KM, Dennis JH, Bass HW (2014). Differential nuclease sensitivity profiling of chromatin reveals biochemical footprints coupled to gene expression and functional DNA elements in maize. Plant Cell.

[42] Du Z, Zhou X, Ling Y, Zhang Z, Su Z. agriGO: a GO analysis toolkit for the agricultural community. Nucleic Acids Research. 2010;38(Web Server issue):W64–70. 10.1093/nar/gkq310 PMC2896167 20435677

[43] Trapnell C, Roberts A, Goff L, Pertea G, Kim D, Kelley DR, Pimentel H, Salzberg SL, Rinn JL, Pachter L (2012). Differential gene and transcript expression analysis of RNA-seq experiments with TopHat and cufflinks. Nat Protoc.

[44] Chandran AKN, Bhatnagar N, Kim B, Jung K-H (2016). Genome-wide identification and analysis of rice genes to elucidate morphological agronomic traits. Journal of Plant Biology.

[45] Basehoar AD, Zanton SJ, Pugh BF (2004). Identification and distinct regulation of yeast TATA box-containing genes. Cell.

[46] Civan P, Svec M (2009). Genome-wide analysis of rice (Oryza Sativa L. subsp. japonica) TATA box and Y patch promoter elements. Genome.

[47] Chen K, Xi Y, Pan X, Li Z, Kaestner K, Tyler J, Dent S, He X, Li W. DANPOS: Dynamic analysis of nucleosome position and occupancy by sequencing. Genome Res. 2013;23(2):341–51. 10.1101/gr.142067.112 PMC3561875 23193179

[48] Madsen JG, Schmidt SF, Larsen BD, Loft A, Nielsen R, Mandrup S (2015). iRNA-seq: computational method for genome-wide assessment of acute transcriptional regulation from total RNA-seq data. Nucleic Acids Res.

[49] Wippo CJ, Krstulovic BS, Ertel F, Musladin S, Blaschke D, Sturzl S, Yuan GC, Horz W, Korber P, Barbaric S (2009). Differential cofactor requirements for histone eviction from two nucleosomes at the yeast PHO84 promoter are determined by intrinsic nucleosome stability. Mol Cell Biol.

[50] Zhou J, Jiao F, Wu Z, Li Y, Wang X, He X, Zhong W, Wu P. OsPHR2 is involved in phosphate-starvation signaling and excessive phosphate accumulation in shoots of plants. Plant Physiol 2008;146(4):1673–1686. 10.1104/pp.107.111443 PMC2287342 18263782

[51] Small EC, Xi L, Wang JP, Widom J, Licht JD (2014). Single-cell nucleosome mapping reveals the molecular basis of gene expression heterogeneity. Proc Natl Acad Sci U S A.

[52] Wang J, Zhuang J, Iyer S, Lin X, Whitfield TW, Greven MC, Pierce BG, Dong X, Kundaje A, Cheng Y (2012). Sequence features and chromatin structure around the genomic regions bound by 119 human transcription factors. Genome Res.

[53] Snyder Matthew W, Kircher M, Hill Andrew J, Daza Riza M, Shendure J. Cell-free DNA comprises an in vivo nucleosome footprint that informs its tissues-of-origin. Cell. 2016;164(1–2):57–68. 10.1016/j.cell.2015.11.050 PMC4715266 26771485

[54] Ulz P, Thallinger GG, Auer M, Graf R, Kashofer K, Jahn SW, Abete L, Pristauz G, Petru E, Geigl JB (2016). Inferring expressed genes by whole-genome sequencing of plasma DNA. Nat Genet.

[55] Jiang C, Pugh BF (2009). Nucleosome positioning and gene regulation: advances through genomics. Nat Rev Genet.

[56] Langmead B, Trapnell C, Pop M, Salzberg SL (2009). Ultrafast and memory-efficient alignment of short DNA sequences to the human genome. Genome Biol.

[57] Ramírez F, Ryan DP, Grüning B, Bhardwaj V, Kilpert F, Richter AS, Heyne S, Dündar F, Manke T (2016). deepTools2: a next generation web server for deep-sequencing data analysis. Nucleic Acids Res.

[58] Benjamini Y, Speed TP (2012). Summarizing and correcting the GC content bias in high-throughput sequencing. Nucleic Acids Res.

[59] Shen L, Shao N, Liu X, Nestler E (2014). Ngs.Plot: quick mining and visualization of next-generation sequencing data by integrating genomic databases. BMC Genomics.

[60] Robinson JT, Thorvaldsdóttir H, Winckler W, Guttman M, Lander ES, Getz G, Mesirov JP (2011). Integrative genomics viewer. Nat Biotechnol.

[61] Quinlan AR, Hall IM (2010). BEDTools: a flexible suite of utilities for comparing genomic features. Bioinformatics.

[62] Kim D, Pertea G, Trapnell C, Pimentel H, Kelley R, Salzberg SL (2013). TopHat2: accurate alignment of transcriptomes in the presence of insertions, deletions and gene fusions. Genome Biol.

[63] Zhang W, Wu Y, Schnable JC, Zeng Z, Freeling M, Crawford GE, Jiang J (2012). High-resolution mapping of open chromatin in the rice genome. Genome Res.

[64] Jones JB. Laboratory guide for conducting soil tests and plant analysis: CRC Press; 2001.

[65] Ames BN (1966). Assay of inorganic phosphate, total phosphate and phosphatases. Methods Enzymol.

