# Supplementary material for: Rice nucleosome patterns undergo remodeling coincident with stress-induced gene expression
Source: BMC Genomics. 2018 Jan 26;19:97. doi: 10.1186/s12864-017-4397-8 (PMC5787291; doi:10.1186/s12864-017-4397-8)
Supplement: Supplementary file 1 — Figure S1. Reproducibility of MNase-seq and RNA-seq. C1, control replicate 1; C2, control replicate 2; P1, −Pi replicate 1, P2, −Pi replicate 2. (A) Clustered heatmap of mapped MNase-seq samples with Spearman correlation coefficient (ρ). The distances among sample pairs are determined as 1-ρ. (B) Clustered heatmap of mapped rmRNA-seq samples with Pearson correlation coefficient (r). The distances among sample pairs are determined as 1-r. (PDF 161 kb) [file 12864_2017_4397_MOESM1_ESM.pdf]

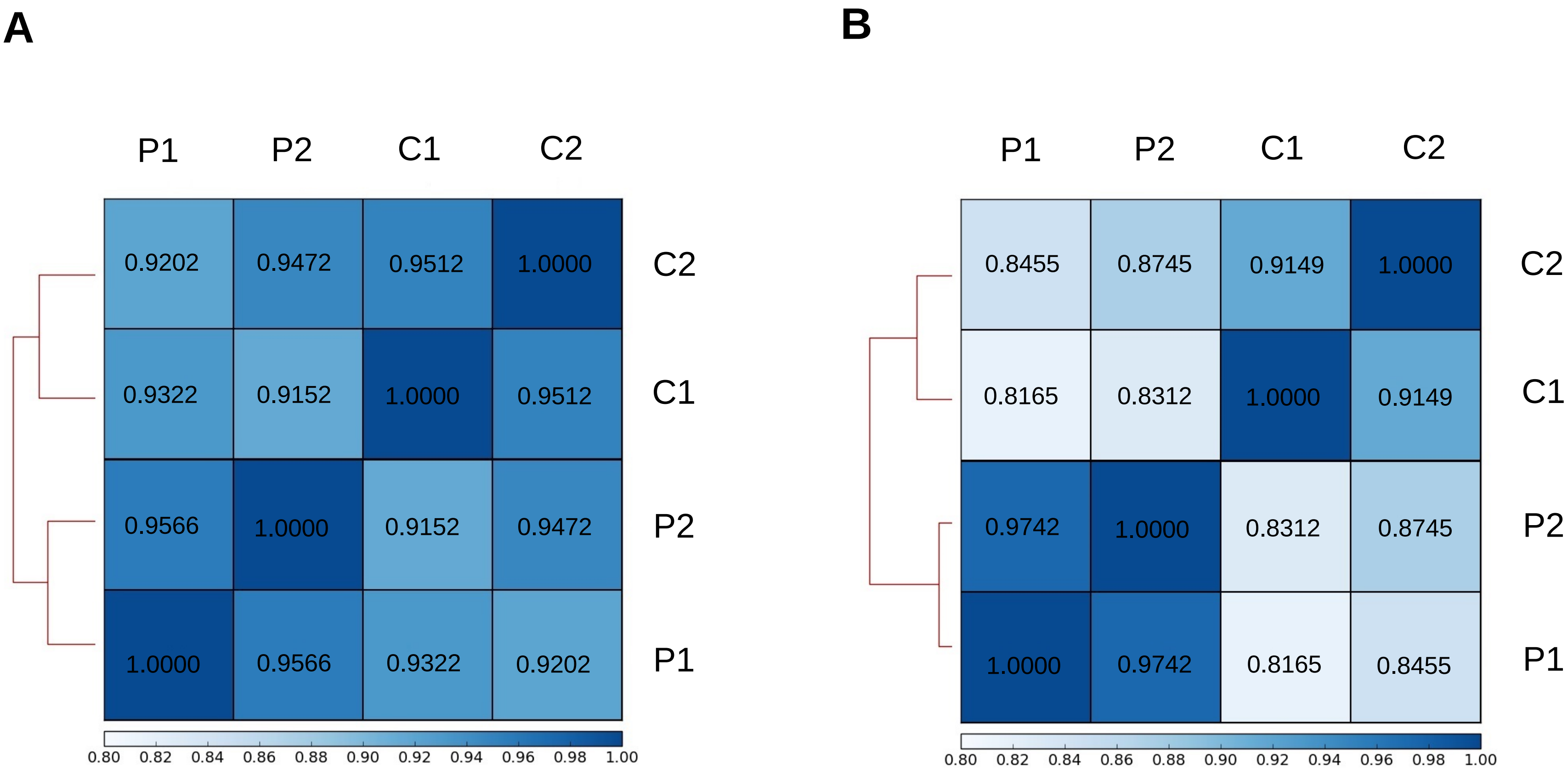


**Figure S1 Reproducibility of MNase-seq and RNA-seq.** C1, control replicate 1; C2, control replicate 2; P1, −Pi replicate 1, P2, −Pi replicate 2. (A) Clustered heatmap of mapped MNase-seq samples with Spearman correlation coefficient (*ρ*). The distances among sample pairs are determined as 1-*ρ*. (B) Clustered heatmap of mapped rmRNA-seq samples with Pearson correlation coefficient (*r*). The distances among sample pairs are determined as 1-*r*.
