# Supplementary material for: Rice nucleosome patterns undergo remodeling coincident with stress-induced gene expression
Source: BMC Genomics. 2018 Jan 26;19:97. doi: 10.1186/s12864-017-4397-8 (PMC5787291; doi:10.1186/s12864-017-4397-8)
Supplement: Supplementary file 2 — Figure S2. Nucleosome patterns across the transcription termination site and gene body of rice genes. Regions 1000 bp upstream and downstream of the transcription termination site (TTS or 3′ boundary of the element), and gene body (GB, from TSS (5′ boundary) to TTS (3′ boundary) of a gene) were used to plot MNase-seq density under control conditions (A and B) and control and –Pi (C and D). (A) MNase-seq density for all rice genes across the TTS under control conditions. (B) MNase-seq density for all rice genes across the GB under control conditions. (C) MNase-seq density of PCG across the TTS from 24-h control and –Pi rice shoots. (D) MNase-seq density of PCG across the GB from 24-h control and –Pi rice shoots. (PDF 77 kb) [file 12864_2017_4397_MOESM2_ESM.pdf]

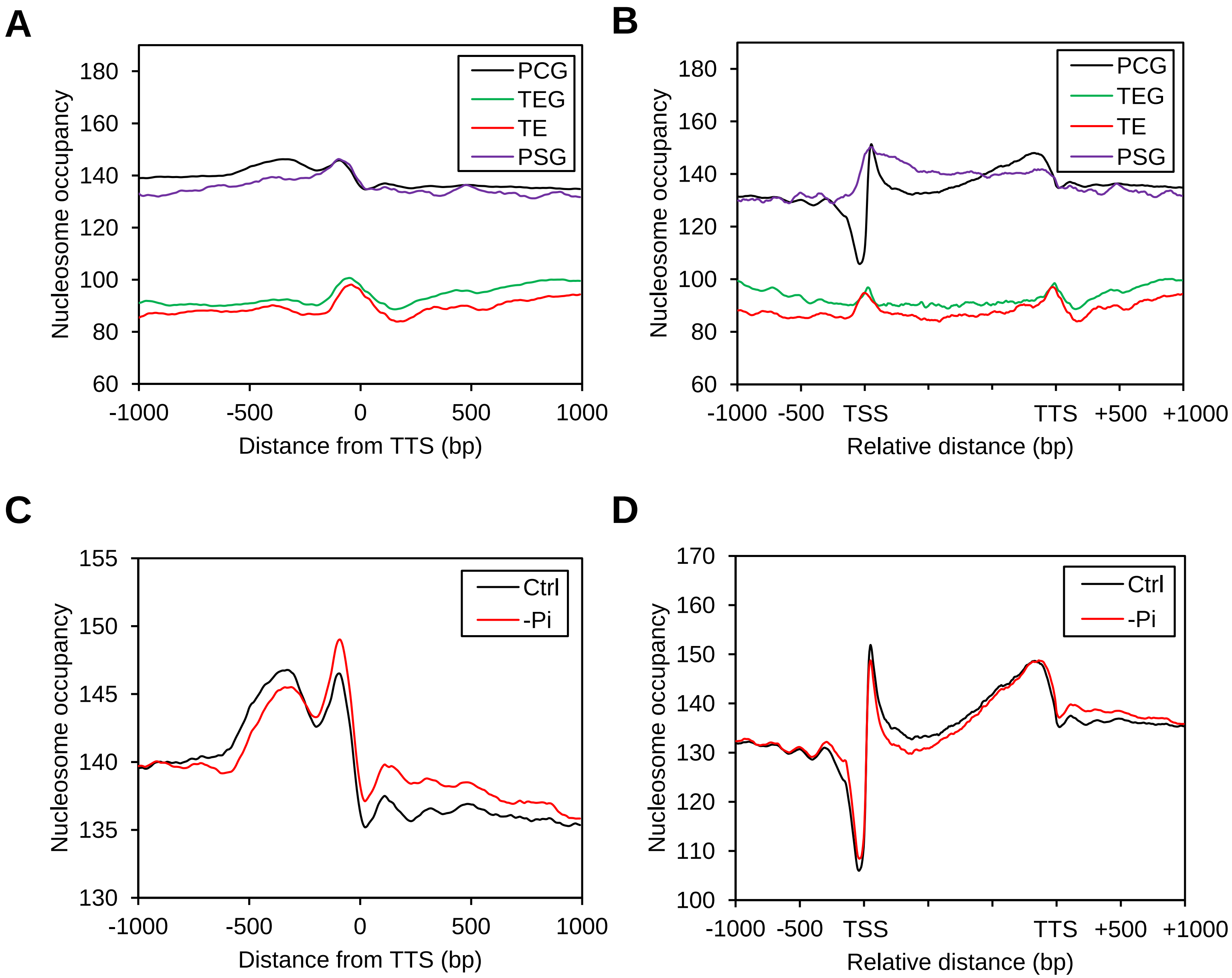

Figure S2 Nucleosome patterns across the transcription termination site and gene body of rice genes. Regions 1,000 bp upstream and downstream of the transcription termination site (TTS or 3' boundary of the element), and gene body (GB, from TSS (5' boundary) to TTS (3' boundary) of a gene) were used to plot MNase-seq density under control conditions (A and B) and control and –Pi (C and D). (A) MNase-seq density for all rice genes across the TTS under control conditions. (B) MNase-seq density for all rice genes across the GB under control conditions. (C) MNase-seq density of PCG across the TTS from 24-hour control and –Pi rice shoots. (D) MNase-seq density of PCG across the GB from 24-hour control and –Pi rice shoots.
