# Supplementary material for: Rice nucleosome patterns undergo remodeling coincident with stress-induced gene expression
Source: BMC Genomics. 2018 Jan 26;19:97. doi: 10.1186/s12864-017-4397-8 (PMC5787291; doi:10.1186/s12864-017-4397-8)
Supplement: Supplementary file 3 — Figure S3. Correlations between nucleosome patterns and gene expression. (A) Heatmap of MNase-seq density of PCG sorted by their expression level under control conditions from RNA-seq analysis of the same tissue (1st highest, 5th lowest). The vertical line in the middle of the heatmap indicates the TSS. (B) Average plot of the same data with genes grouped by their expression levels. (PDF 65 kb) [file 12864_2017_4397_MOESM3_ESM.pdf]

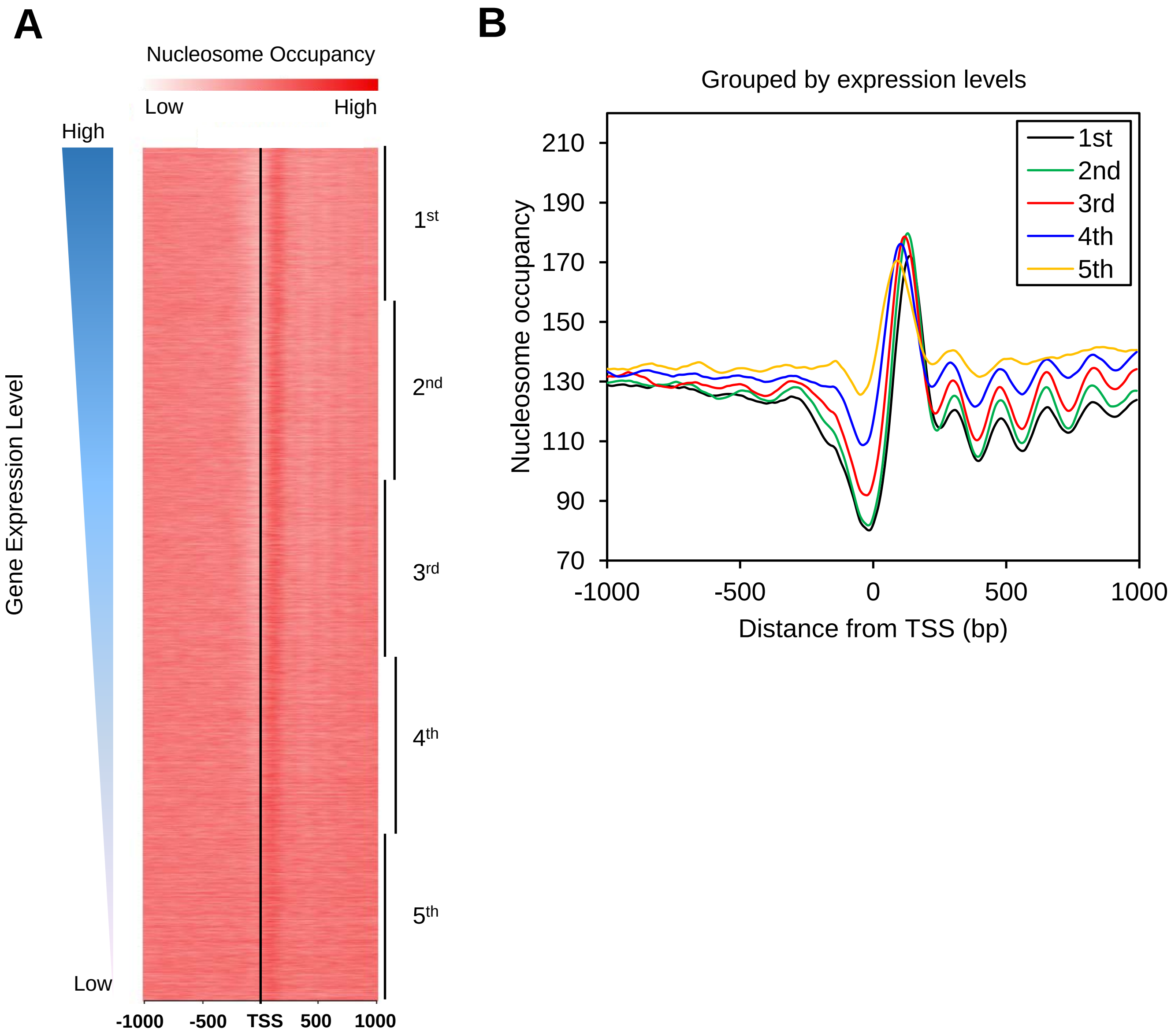

Figure S3 Correlations between nucleosome patterns and gene expression. (A) Heatmap of MNase-seq density of PCG sorted by their expression level under control conditions from RNA-seq analysis of the same tissue (1st highest, 5th lowest). The vertical line in the middle of the heatmap indicates the TSS. (B) Average plot of the same data with genes grouped by their expression levels.
