# Supplementary material for: Rice nucleosome patterns undergo remodeling coincident with stress-induced gene expression
Source: BMC Genomics. 2018 Jan 26;19:97. doi: 10.1186/s12864-017-4397-8 (PMC5787291; doi:10.1186/s12864-017-4397-8)
Supplement: Supplementary file 4 — Dataset 1. GO term enrichment for six clusters of genes. (ZIP 1029 kb) [file 12864_2017_4397_MOESM4_ESM.zip]

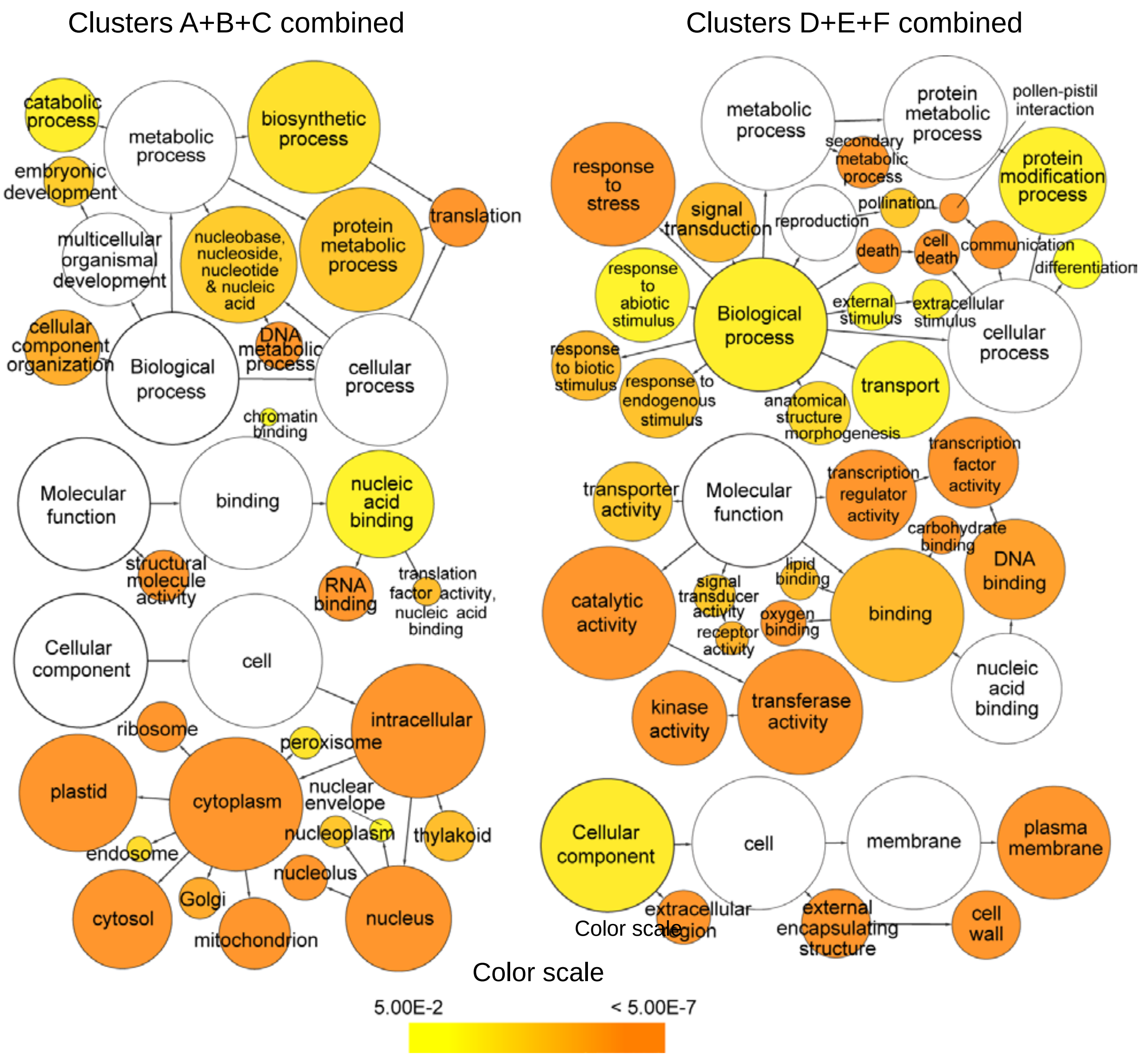

Figure S4 Significantly enriched GO terms for clusters ABC (type I gene) and DEF (type II gene). The color of the node represents the corrected *p*-value with a color scale ranging from yellow (corrected *p*-value=0.05) to dark orange (corrected *p*-value= 5×10^{-7}).
