# Supplementary material for: Rice nucleosome patterns undergo remodeling coincident with stress-induced gene expression
Source: BMC Genomics. 2018 Jan 26;19:97. doi: 10.1186/s12864-017-4397-8 (PMC5787291; doi:10.1186/s12864-017-4397-8)
Supplement: Supplementary file 5 — Figure S4. Significantly enriched GO terms for clusters ABC (type I gene) and DEF (type II gene). The color of the node represents the corrected p-value with a color scale ranging from yellow (corrected p-value = 0.05) to dark orange (corrected p-value = 5 × 10^−7^). (PDF 443 kb) [file 12864_2017_4397_MOESM5_ESM.pdf]

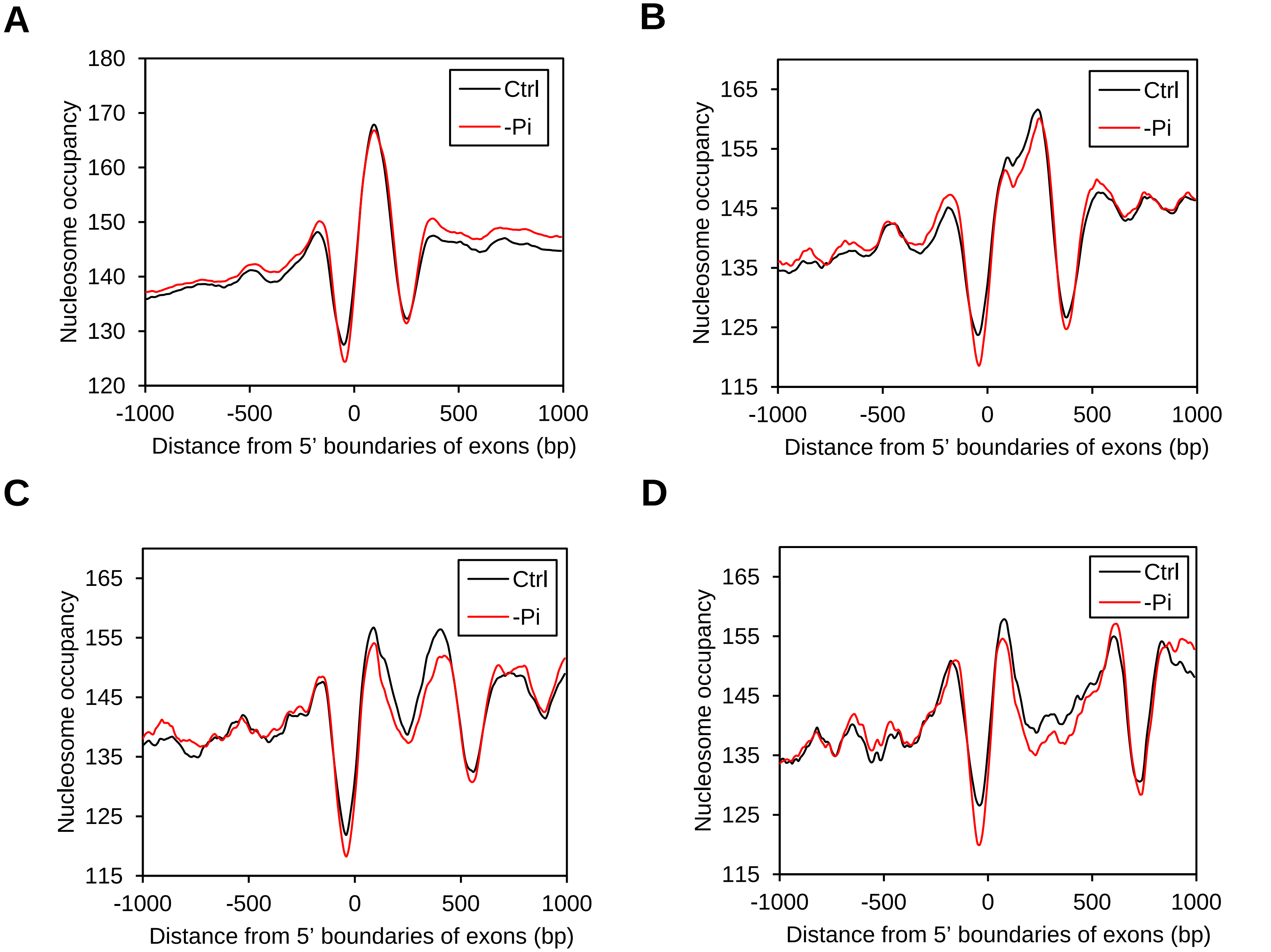

Figure S5 Changes in nucleosome occupancy at the exons of rice genes in response to phosphate starvation. MNase-seq density of exons grouped according to their length: (A) 170−240 bp; (B) 315−350 bp; (C) 480−550 bp (D) 645−715 bp under control and −Pi conditions. Plots are centered at the 5’ boundaries of the exons.
