# Supplementary material for: Rice nucleosome patterns undergo remodeling coincident with stress-induced gene expression
Source: BMC Genomics. 2018 Jan 26;19:97. doi: 10.1186/s12864-017-4397-8 (PMC5787291; doi:10.1186/s12864-017-4397-8)
Supplement: Supplementary file 6 — Figure S6. Changes of total phosphorus and inorganic phosphate concentrations in response to phosphate starvation. All values are the mean ± standard error of the mean; n = 3 biological replicates with 3 technical repeats each. DW, dry weight; FW, fresh weight. (A) Total phosphorus (P) concentrations for shoots of 5-week-old seedlings grown under full nutrient (Ctrl) and Pi-starvation (−Pi) conditions. (B) Total P concentrations for roots of 5-week-old seedlings grown under full nutrient (Ctrl) and Pi-starvation (−Pi) conditions. (C) Inorganic phosphate (Pi) concentrations for shoots of 5-week-old seedlings grown under full nutrient (Ctrl) and Pi-starvation (−Pi) conditions. (D) Pi concentrations for roots of 5-week-old seedlings grown under full nutrient (Ctrl) and Pi-starvation (−Pi) conditions. (PDF 38 kb) [file 12864_2017_4397_MOESM6_ESM.pdf]

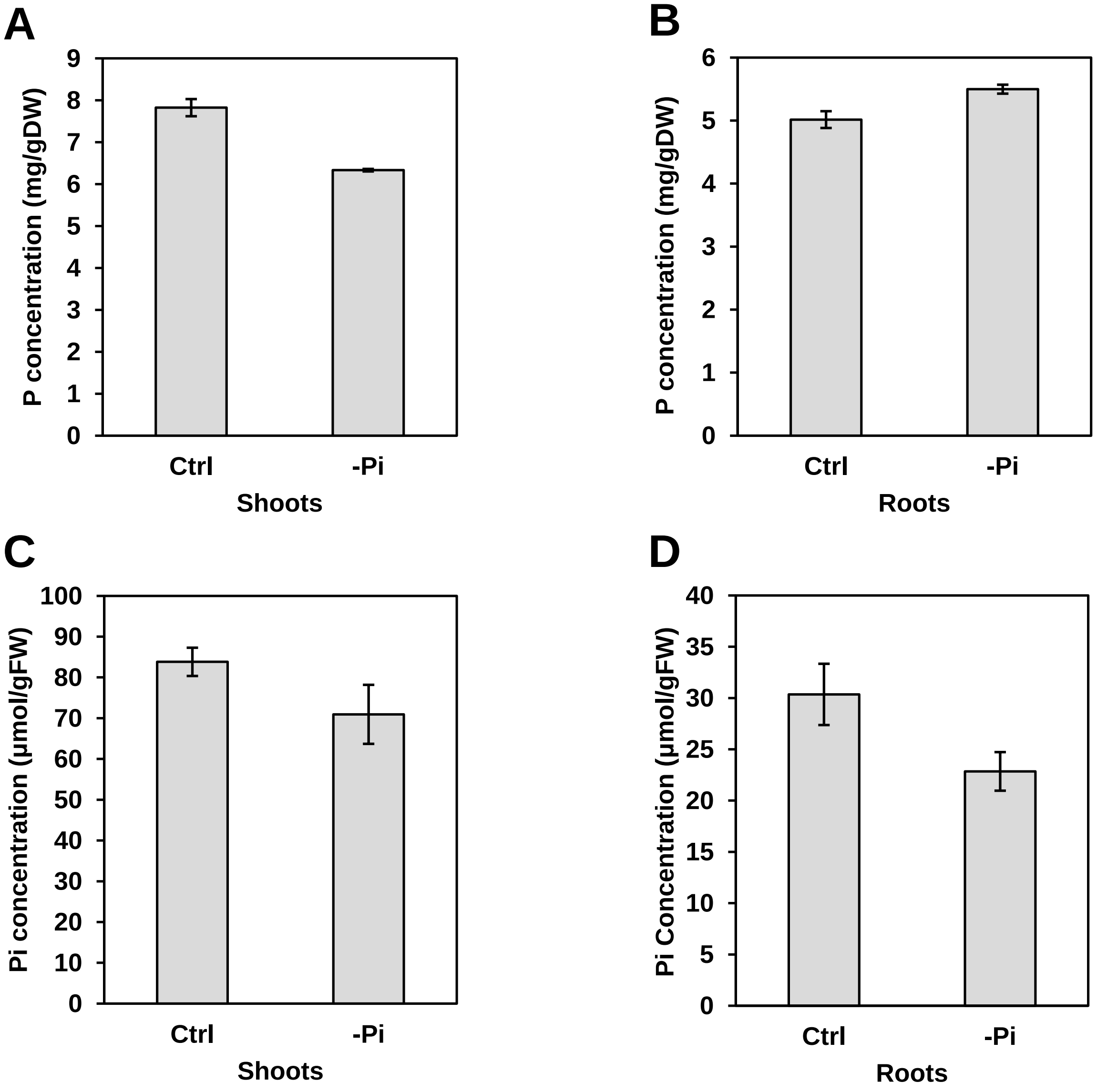

Figure S6 Changes of total phosphorus and inorganic phosphate concentrations in response to phosphate starvation. All values are the mean ± standard error of the mean; n=3 biological replicates with 3 technical repeats each. DW, dry weight; FW, fresh weight. (A) Total phosphorus (P) concentrations for shoots of 5-week-old seedlings grown under full nutrient (Ctrl) and Pi-starvation (−Pi) conditions. (B) Total P concentrations for roots of 5-week-old seedlings grown under full nutrient (Ctrl) and Pi-starvation (−Pi) conditions. (C) Inorganic phosphate (Pi) concentrations for shoots of 5-week-old seedlings grown under full nutrient (Ctrl) and Pi-starvation (−Pi) conditions. (D) Pi concentrations for roots of 5-week-old seedlings grown under full nutrient (Ctrl) and Pi-starvation (−Pi) conditions.
